# Post-operative anticoagulation therapy after knee or hip replacement: The role of patients’ preferences in selection of therapy

**DOI:** 10.21203/rs.3.rs-7289754/v1

**Published:** 2025-08-06

**Authors:** Leslie A. Lenert, Dmitry Scherbakov, Azza Shaoibi, Brian Neelon, Amy Reynolds, Laura Kernan, Carol A. Lambourne, Vincent D. Pellegrini

**Keywords:** treatment satisfaction, anticoagulants, conjoint analysis, aspirin, warfarin, rivaroxaban

## Abstract

**Objectives:**

Patients who receive hip or knee replacement surgery should be anticoagulated to prevent thrombosis-related events, such as pulmonary embolism. The Pulmonary Embolism Prevention after Hip and Knee Replacement (PEPPER) is a large pragmatic trial studying which anticoagulant (aspirin, warfarin, or rivaroxaban) is optimal. As an adjunct to this study, we examined the role of patients’ preferences in individual tailoring of therapy.

**Methods:**

We constructed a multimedia conjoint analysis (CA) survey based on anticoagulants' beneficial and adverse effects at the expected probabilities being studied in the PEPPER trial. We recruited 212 hip and knee post-surgery patients at the Medical University of South Carolina who were eligible for the PEPPER trial and studied their preferences one to seven months after surgery. K-means clustering was used to characterize heterogeneity in patients’ preferences.

**Results:**

Across the studied population, expected risks of major adverse effects (bleeding, venous thrombosis, and pulmonary embolism) were, on average, rated as being of similar importance, with somewhat greater weight being placed on avoiding risks of pulmonary embolism. However, few patients had values near the average for the population, with patients grouping in three distinct, minimally-overlapping segments or phenotypes: Thrombosis-focused values (aligns with rivaroxaban treatment), Balanced values (bleeding and thrombosis-focus (aligns with aspirin)), and “Out-of-pocket-cost focused values (aligns with aspirin or warfarin).

**Conclusions:**

In the post-knee/hip replacement setting, a CA survey revealed that patients value the risks and benefits of anticoagulation differently, falling into three distinct phenotypes that have implications for the individualization of therapy. Providers should tailor post-operative anticoagulation to patients’ preferences.

## Introduction

In the clinical context of knee and hip replacement surgeries, the prevention of venous thromboembolism (VTE) using anticoagulants is the standard of care due to the high risk of VTE postoperatively. Studies have shown that VTE is a frequent and potentially fatal complication after these surgeries, with a significant percentage of patients developing deep venous thrombosis without adequate prophylaxis^1, 2^. The prevalence of asymptomatic deep vein thrombosis is higher after knee replacement initially, but symptomatic venous thromboembolism is also likely to occur after hip replacement^3^. There are several different options for post-operative anticoagulation: use of full-dose aspirin as a platelet aggregation inhibitor; use of Warfarin, a well-studied vitamin K-dependent factor synthesis inhibitor that requires monitoring and adjustment, and use of a direct inhibitor of factor IIa or Xa, such as rivaroxaban which inhibits Xa and offers intensive anticoagulation without the need for monitoring. More intensive anti-coagulation has adverse effects: increased risks of major bleeding, particularly into the joint replacement, which could lead to infection and the requirement for joint replacement^4^.

The optimal choice of anticoagulant and duration of therapy post-hip and knee replacement surgeries is not known and is the subject of a very large pragmatic randomized trial funded by the Patient-Centered Outcomes Research Institute called the Pulmonary Embolism Prevention after Hip and Knee Replacement Trial (PEPPER)^5^. In this trial, hip or knee replacement patients are randomly assigned to either initial Warfarin, aspirin, or rivaroxaban prophylaxis. They are then followed for six months to assess outcomes related to thromboembolism, adverse effects of bleeding, and functional status postoperatively.

In a non-randomized trial setting, it is well-known that anticoagulation should be individualized based on the patient's risk factors, bleeding tendencies, and the need for continued thromboprophylaxis^6^. However, there is currently little data on how patients’ preferences should impact decision-making. Do patients value the different kinds of risk differently (bleeding versus thromboembolism)? Are costs such as co-pays for medication an essential factor in patient decision-making? Is the inconvenience of warfarin monitoring, even over a short period, a significant factor? Adverse events occur at low-frequency rates. Will patients judge them as important, or will they assume negligible risks relative to certain direct costs of anticoagulants? To address these issues, we developed a multimedia survey that described to patients the different potential outcomes of post-surgical anticoagulation choices and measured their preferences using a conjoint analysis technique. To validate survey results, we also measured patients’ experiences with post-operative anticoagulation therapy and attempted to correlate satisfaction and treatment with measurements of their preferences.

## Methods

### Survey development

Working from the approved PEPPER trial protocol, we identified the adverse events that the trial was powered to detect differences across treatments. These focused on the risk of deep venous thrombosis, the risk of pulmonary embolism, the risk of bleeding leading to artificial joint failure and replacement, and financial toxicity. We also reviewed the literature and study design documents to estimate the probabilities of the rare events and then created composite descriptions of the events. These descriptions were first created in text form and then transformed into scripts that were subsequently turned into animated graphics illustrating each problem. The scripts and the presentations were reviewed by the lead surgeon in the trial and the patient advisory board for the trial. Input was solicited at each stage of production to ensure materials were not only medically valid but highly comprehensible to patients.

**Figure 1:**
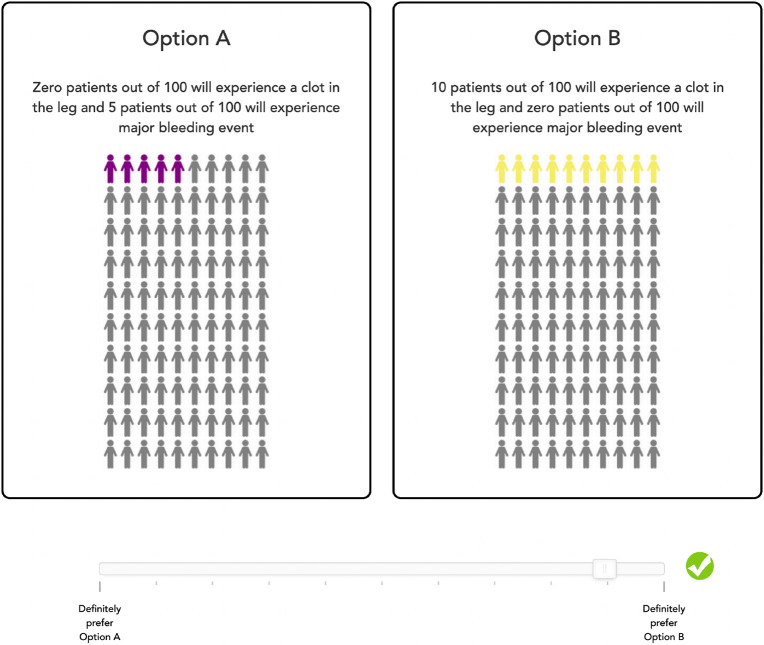
Conjoint analysis question from the preferences survey.

To display the probability of different adverse effects, we used a standardized approach where panels of stick figures were used in 10X10 grids, with shading of the figures in sequence to show the probability of a specific type of adverse event. Links to the video descriptions of each event were embedded in the panels about the displays. In ratings, participants clicked on a scale to indicate their strength of preference for one state over another. The further to the right or left, the stronger their preference (Fig. 1). The strength of preference was captured as a continuous measure based on the distance from the midpoint of a displayed line.

This preference survey followed a pairwise partial profile conjoint analysis design described in a previous publication^7^. The pairwise choices of the following attributes/events of anticoagulant drugs were displayed:

- A copayment of $200 for medication
- No copayment
- Repeated blood tests
- No blood tests required
- Risk of major bleeding
- No risk of major bleeding
- Minor bleeding risk
- No risk of minor bleeding
- Risk of a clot in the lung
- No risk of clots in the lung
- Risk of a clot in the leg
- No risk of a clot in the leg

These factors and levels were chosen based on the design of the PEPPER study to support subsequent inference of overall effectiveness. The PEPER patient advisory panel (see acknowledgments) also provided input into the significance of side effects and the specific descriptions. In addition, at the start of the survey, participants were asked to rank the different types of adverse effects or financial impacts by arranging items in their preferred order (vertically).

A live version of the instrument can be found at https://preferences.musc.edu/surveys/PEPPER/take/ID, where ID should be replaced with a unique random string (e.g., TEST1234). After completing the preference survey, patients completed the Anti-Clot Treatment Scale survey (ACTS)^8^, a well-validated instrument with three scales: one for satisfaction with the benefits of anticoagulation, one for dissatisfaction with potentially harmful or inconvenient aspects of therapy, and an overall measure. The patient preference and satisfaction surveys were administered to patients at the same time, approximately eight weeks after surgery and 4 weeks after completion of anticoagulation prophylaxis. Patients who were in the PEPPER study who received surgery at about the same time were included in the study supplement recruitment from the trial population.

We then collected and analyzed the results of the preference survey and treatment satisfaction in a cross-sectional study of patients. We use linear regression and the results of the ACTS survey to evaluate if there is a relationship between treatment received, patient’s initial preference cluster, and their final satisfaction with the treatment.

In summary, the following data was captured and analyzed:

1. The ordered importance of each of the attributes for each patient was captured;
2. Rated different combinations of attributes at a displayed probability of risk, comparing two attributes at a time.
3. Fit an overall model of preferences for each patient.
4. Computed the relative importance of each factor for each patient and scaled the relative importance from 0 to 1, where 1 is the most important attribute for the patient;
5. Used k-means clustering to segment patients into different groups, identifying the optimal solution for representing the population.
  - Using principal components analysis, we identified orthogonal factors that capture the most variance in data to plot the observations on a two-dimensional diagram.
  - The number of clusters (k) was determined using the Silhouette method.
6. We added information on the patient's actual treatment, classifying care as concordant (aligned with) or discordant (not aligned) with the preference cluster to which the patient was assigned. Concordance was determined by the importance of different types of adverse effects in the patient’s overall preference weights and anticipated effects of the three therapies under evaluation (aspirin, warfarin, and rivaroxaban).

### Patient recruitment

Participants were recruited from the population of patients undergoing elective knee or hip replacement at the Medical University of South Carolina between 2020-06-17 and 2023-10-07 who were eligible for PEPPER. Patients who had expressed interest in the study during recruitment for the PEPPER trial or who had undergone surgery, met general eligibility criteria, and were awaiting their first post-surgical follow-up visit (two to six weeks after surgery) were contacted by MyChart, email, or phone calls and were recruited for participation in the study. Patients who agreed had their eligibility confirmed and consented to study participation. They then completed the survey via telehealth appointment or in person before meeting with their surgeon during the follow-up visit under the supervision of the research assistant. Patients were not compensated for taking part in this study.

### Survey administration

Patients completing the survey via telehealth either 1) met online virtually using Doxy.me telehealth software and then completed the survey under the remote observation of the research assistant or 2) met on the telephone and simultaneously used a shared website to complete the survey. Patients completing the survey in person did so in the presence of the research assistant using a laptop computer in the follow-up clinic prior to their visit. The research assistant helped the patient respond to survey questions as needed with carefully controlled prompting. All patients completed the preferences survey first and then the anticoagulation satisfaction survey. Patients responding to the survey remotely were allowed to complete the satisfaction survey without supervision once the preferences part was completed to reduce resource requirements for the study.

## Results

1001 patients were contacted by direct outreach methods using electronic health record data, and another 472 patients were contacted after referral from the PEPPER trial. Of these, 212 agreed to participate in the study, of which 211 completed the preferences survey, and 192 out of 211 completed the post-treatment anticoagulation satisfaction survey. Surveys were completed at a median of 60 days (IQR: 23–209) after surgery (SD = 136.3). There were 8 crossovers (drug changes) in PEPPER patients, of which 3 occurred after hospital discharge, and the other 5 occurred before or during hospitalization. Demographics for the final list of 192 patients are provided in Table 1.

**Figure 2:**
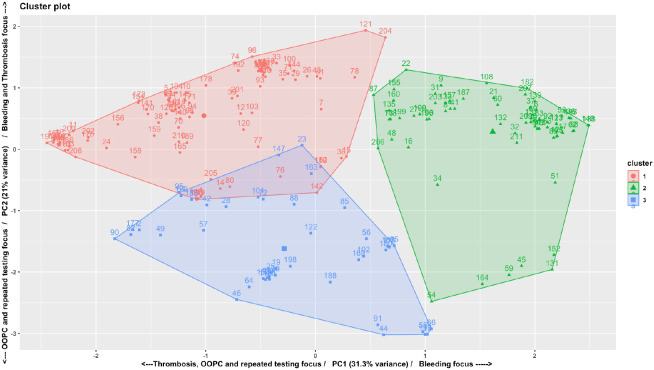


None of the patients reported experiencing the outcomes described in the survey. When looking at the ranking data for the least desirable outcomes, pulmonary embolism, followed by major bleeding and deep venous thrombosis, were the worst (probability-adjusted) outcomes. 58.1%, 31.4%, and 5.97% of patients rated pulmonary embolism scenario, major bleeding scenario, and deep venous thrombosis scenario, respectively, as the worst outcome. Table 2 shows the analysis of the conjoint analysis survey results. The average (across all patients) importance score for the risk of clot in the lung was the highest (score 0.568), with similar importance of major bleeding and deep venous thrombosis, while the risk of minor bleeding was the lowest (0.208). After the principal components analysis, K-means clustering revealed three distinct clusters (Fig. 1), with the cluster centers plotted on the axes of the two most prominent factors, as displayed in Table 2. We named each cluster based on the dominant factor Thrombosis-focused (n = 90, 46.9%), which rated the importance of pulmonary embolism as most important; Balanced (n = 61, 31.8%), which rated the risk of postoperative bleeding complications as most important but also considered clotting issues; and Out of Pocket Cost (OOPC) focused (n = 41, 21.3%) patients who focused on the copay amount. We note that few patients had preferences near the “average” value, indicating that the overall population mean is not representative of the population (see Fig. 2). There was no difference in demography across clusters (See Supplemental Table 1).

Patients’ responses from the satisfaction survey are summarized in Table 3. Additional ANOVA tests showed that there were no statistically significant differences between clusters in perceived benefits or burdens, but there was a trend toward the overall satisfaction of therapy (calculated as the difference between perceived benefits and burdens) being different and potentially lower in the OOPC-focused group. ANOVA test did not show the difference in the locus of control for decision-making between clusters—therefore, clustering was not a proxy for preferred decision-making style in clinical settings.

The results of linear regression analysis evaluating the impact of treatment received on the overall perceived burdens scale depending on the initial preference cluster (with Thrombosis-focused patients on aspirin being the reference group) show that treatment satisfaction varies among clusters (Table 4). The statistically significant associations show that OOPC-focused patients had the highest perceived burdens if treated with warfarin (M = 6.3, SD = 11.9; p = 0.013). At the same time, thrombosis-focused patients reported similarly high burdens when treated with warfarin (M = 6.1, SD = 3.4; p = 0.011) and rivaroxaban (M = 5.8, SD = 5.3; p = 0.002), while reporting the lowest burdens with aspirin (M = 1.9, SD = 3.4; p = 0.005). This provides some evidence that cluster membership is associated with experienced satisfaction with treatment.

## Discussion

The relative risks and effectiveness of each approach for anticoagulation after hip or knee replacement are being studied in the PEPPER trial. Once known, this will certainly inform the choice of anticoagulant. But is knowledge of relative risks and benefits enough? Our study sheds new light on the role of patient preferences in the interpretation of risks. Using advanced digital health methods, including multimedia description of outcomes and computerized discrete choice elicitation of preferences, we found three distinct segments in a population of patients undergoing knee or hip replacement surgery – what might be called *preference phenotypes*^9, 10^, where members of this group weighted attributes of therapy above all others, making this factor potentially dominant in decision making for the individual. The outcomes were described using realistic video materials, and patients considered their importance at realistic probabilities of occurrence. The results suggest that the choice of anticoagulant for patients should be informed by patient preferences. Patients who rate pulmonary embolism as the worst event should be treated with the anticoagulant with the greatest potency for its prevention. We suspect from prior work in other settings, such as atrial fibrillation, but have not yet observed in the PEPPER trial, that the lowest risk of pulmonary embolism would occur with a factor Xa inhibitor treatment. If the trial bears this out, then for these patients, Rivaroxaban (or other similar factor Xa inhibitors) would be the optimal anticoagulant. For those concerned with bleeding, we suspect, but do not yet know from PEPPER, that the risk of bleeding complications is lowest in the aspirin group. And, for those concerned with cost, we can know, based on insurance status and copays, if either warfarin or aspirin offers a cost-effective approach for anticoagulation that meets a patient’s financial needs. Determining which preference phenotype a patient has requires the completion of a computer survey, a task that takes about 20–30 minutes. However, the use of Bayesian methods may allow the determination of cluster membership with only one or two questions^7^.

Prior work on measurement of preferences for anticoagulation therapy has focused on looking at the *average, rather than segmenting the population into preference phenotypes.* The most important factor for choice of therapy, across many previous studies, was its effectiveness in preventing thrombosis-related complications such as a life-threatening PE. Haac et al. studied the preferences of trauma patients for anticoagulation care—a similar setting with temporary anticoagulation^11^. Their findings, similar to ours, suggested that, on average, avoidance of thromboembolism was a more important factor to patients in drug choice, with the risk of bleeding also being an important factor. There have been several studies of patients’ preferences in the settings of long-term anticoagulation for atrial fibrillation and chronic thromboembolism prevention in cancer care. These studies confirm our knowledge about what is important on average. Abraham et al. studied patients’ preference for longer-term anticoagulation with adaptive conjoint analysis^12^. Prevention of thrombosis was, again, at the top priority for patients. Concordance of treatment with patients’ preferences may have improved their medication adherence, as measured by refills. Lane and colleagues studied the preferences of atrial fibrillation and found thrombosis prevention was the most important factor overall, with bleeding risk a close second^13^. Gonzales-Rojas performed a conjoint analysis survey in Spanish atrial fibrillation patients with similar methods and findings^14^. Similar findings have been seen in patients with atrial fibrillation in an Australian study^15^ and a Chinese study^16^.

This study focused on the segmentation of the population rather than looking at an “average” (population-wide) view of preferences. We also found bleeding and thrombosis risk to be important factors; some patients held thrombosis to be the most important factor, and others took a balanced view of risk (bleeding and thrombosis). In addition, a novel result of this study is the finding of a subgroup of patients where prevention of (low risks of) thromboembolism or bleeding did not take precedence. This group of patients, which is equally large as the other two, is most concerned about the prevention of a new type of toxicity, the *financial toxicity*, of the drug regimen^17^. The certain costs of Xa inhibitors, which for many patients could be hundreds of dollars per month if not fully covered by insurance, were more important than small differences in low risks of either thromboembolism or bleeding. Financial toxicity is not commonly discussed in anticoagulation decision-making, but it is of growing importance in cancer, diabetes, and heart failure^18-20^.

The perception of risk may be an important factor in this analysis. We used best practices for an unbiased display of risks in this study. But, because the risks were very low, some patients may see them as negligible, discounting the importance of the event. In this situation, shared decision-making may be particularly important, with provider input on patient-specific risks.

Which of the three groups to which a patient belongs is easily identified through the completion of a 20- 30-minute computer questionnaire, which might be done online ahead of a visit planning surgery. Because of the heterogeneity of preferences, post-operative anticoagulation is a situation where optimization of therapy requires close collaboration between the surgeon and the patient, potentially informed by preference elicitation. While discussions on this issue might be time prohibitive in clinical practice, value clarification and categorization, using our questionnaire could make this feasible. Further work in this area is needed to determine this, but the results are promising.

One of the strengths of this study, relative to others, is its use of digital materials and graphic depictions of probabilities to provide clear and understandable descriptions of the outcomes being rated in conjoint analysis. This approach is not widely used in studies of patient preferences due to the complexity of administering the survey and the requirement for a computer-assisted survey approach.

### Limitations

This study has several limitations, particularly as the foundation for future work on shared decision-making for post-operative anticoagulation care. The sample size of our study is relatively modest (n = 192) and limited to a single site. Only about 1 in 7 patients undergoing surgery during the period of the study opted into participation based on contact logs. Our ability to recruit patients into this study was more limited than anticipated because of patients’ advanced age, lack of interest in study participation after the surgery, and other post-operative factors. Preferences were not measured prior to surgery, so we do not know if treatment preferences might be somehow changed by the experience of surgery and treatment with anticoagulants. Additionally, incomplete information on participant socioeconomic status limited our analysis of patient preference cohorts which may be influenced by these social determinants of health. Our sample size was reduced due to logistical issues from the planned size and did not allow us to fully evaluate the effects of preference clusters on satisfaction or to use the more advanced models proposed by Shaobi et al. to fit data on preferences and satisfaction simultaneously to identify clusters^7^. However, we were able to show some association between patients’ preferences, treatment, and their satisfaction with anticoagulation care in concurrent measurements, suggesting that values, as measured by conjoint analysis, are associated with patients’ experiences with therapy.

## Conclusion

In a single-site study using advanced digital media survey techniques, we identified three distinct preference phenotypes in patients that should be considered in postoperative anticoagulation choice. For many patients in our study, the effectiveness of an anticoagulant in preventing thromboembolism is not their most important concern. In some, risks of bleeding and clotting were considered nearly equally important. For others, costs were more important. Providers should share decision-making with patients on the choice of anticoagulant after knee and hip replacement. Further study of conjoint analysis, shaped by digital measurements of preferences, should be considered in helping providers tailor treatment choices to patients’ preferences.

## Figures and Tables

**Table 1:** Patient demographics

		Aspirin	Rivaroxaban	Warfarin	Total
Total N (%)		108 (56.2)	55 (28.6)	29 (15.1)	192
Age, years	Mean (SD)	65.0 (10.2)	63.5 (11.1)	63.4 (12.5)	64.4 (10.8)
Gender	Female	63 (58.3)	36 (65.5)	18 (62.1)	117 (60.9)
	Male	45 (41.7)	19 (34.5)	10 (34.5)	74 (38.5)
	(missing)			1 (3.4)	1 (0.5)
Race	Black or African American	22 (21.8)	8 (15.1)	1 (4.0)	31 (17.3)
	White or Caucasian	79 (78.2)	44 (83.0)	24 (96.0)	147 (82.1)
	Refused to answer		1 (1.9)		1 (0.6)
Ethnicity	Not Hispanic or Latino	100 (92.6)	52 (94.5)	26 (89.7)	178 (92.7)
	Refused to answer		1 (1.8)		1 (0.5)
	(missing)	8 (7.4)	2 (3.6)	3 (10.3)	13 (6.8)
PEPPER participant	No	69 (63.9)	18 (32.7)	5 (17.2)	92 (47.9)
	Yes	39 (36.1)	37 (67.3)	24 (82.8)	100 (52.1)

**Table 2:** Preference clusters identified from conjoint analysis survey data. K-means clustering. 0 means having this risk of the drug is least important for the patient, and 1 – the opposite. The highest value is in bold.

	Cluster name	Risk of minor bleeding	Risk of major bleeding	Risk of pulmonary embolism	Risk of clot in leg	Copay 200	Blood tests required
Overall avg score	-	0.208	0.521	**0.568**	0.565	0.443	0.428
Cluster 1	Thrombosis focu	0.184	0.369	0.819	**0.952**	0.43	0.55
Cluster 2	Balanced focus	0.181	**0.93**	0.439	0.203	0.2	0.15
Cluster 3	OOPC focus	0.301	0.22	0.224	0.286	**0.843**	0.591

**Table 3:** Satisfaction survey data summary table with preference clusters information. ANOVA test for continuous variables, Fisher’s Exact Test for cluster variable.

		aspirin	rivaroxaban	warfarin	p-value
Total N (%)		108 (56.2)	55 (28.6)	29 (15.1)	
Sum of benefits	Mean (SD)	10.7 (4.6)	11.7 (4.3)	10.3 (4.9)	0.301
Sum of burdens	Mean (SD)	2.6 (4.4)	4.5 (4.7)	6.1 (7.4)	0.002
Overall benefits minus burdens	Mean (SD)	8.2 (6.5)	7.3 (6.0)	4.3 (8.8)	0.024
Locus in decision making (-5 – confidence in own decision-making, + 5 – trust their doctor in choice of anticoagulation)	Mean (SD)	0.7 (4.3)	1.2 (4.0)	1.7 (4.2)	0.506
Cluster	Thrombosis focus patients	53 (49.1)	25 (45.5)	12 (41.4)	0.471
	Balanced focus patients	35 (32.4)	19 (34.5)	7 (24.1)	
	OOPC focus patients	20 (18.5)	11 (20.0)	10 (34.5)	

**Table 4:** Linear regression analysis evaluating the impact of treatment received on overall perceived burdens depending on initial cluster.

		Overall burdens Mean (SD)	Linear regression coefficients
Cluster : treatment	Intercept [Thrombosis focused patients : aspirin]	1.9 (3.4)	1.9 (0.58 to 3.30, p = 0.005)
	Thrombosis focus patients : rivaroxaban	5.8 (5.3)	3.90 (1.49 to 6.30, p = 0.002)
	Thrombosis focus patients : warfarin	6.1 (3.4)	4.14 (0.97 to 7.31, p = 0.011)
	Balanced focus patients : aspirin	2.6 (3.2)	0.63 (−1.53 to 2.79, p = 0.567)
	Balanced focus patients : rivaroxaban	3.1 (4.2)	1.16 (−1.49 to 3.81, p = 0.388)
	Balanced focus patients : warfarin	5.7 (4.6)	3.77 (−0.22 to 7.76, p = 0.064)
	OOPC focused patients : aspirin	4.2 (7.4)	2.26 (−0.34 to 4.86, p = 0.089)
	OOPC focused patients : rivaroxaban	3.8 (3.1)	1.87 (−1.41 to 5.16, p = 0.262)
	OOPC focused patients : warfarin	6.3 (11.9)	4.36 (0.94 to 7.77, p = 0.013)

## Data Availability

The datasets generated during and/or analyzed during the current study are available from the corresponding author on reasonable request.
